# Host Response Modulation Therapy in the Diabetes Mellitus—Periodontitis Conjuncture: A Narrative Review

**DOI:** 10.3390/pharmaceutics14081728

**Published:** 2022-08-18

**Authors:** Irina-Georgeta Sufaru, Silvia Teslaru, Liliana Pasarin, Gianina Iovan, Simona Stoleriu, Sorina Mihaela Solomon

**Affiliations:** 1 Department of Periodontology, Grigore T. Popa University of Medicine and Pharmacy, Universitatii Street 16, 700115 Iasi, Romania; 2 Department of Cariology and Restorative Dental Therapy, Grigore T. Popa University of Medicine and Pharmacy, Universitatii Street 16, 700115 Iasi, Romania

**Keywords:** diabetes mellitus, host inflammatory response, host response modulation therapy, oxidative stress, periodontitis

## Abstract

The inflammatory response of the host in periodontitis is the phenomenon that underlies the onset and evolution of periodontal destructive phenomena. A number of systemic factors, such as diabetes mellitus (DM), can negatively affect the patient with periodontitis, just as the periodontal disease can aggravate the status of the DM patient. Host response modulation therapy involves the use of anti-inflammatory and anti-oxidant products aimed at resolving inflammation, stopping destructive processes, and promoting periodontal healing, all important aspects in patients with high tissue loss rates, such as diabetic patients. This paper reviews the data available in the literature on the relationship between DM and periodontitis, the main substances modulating the inflammatory response (nonsteroidal anti-inflammatory drugs, sub-antimicrobial doses of doxycycline, or omega-3 fatty acids and their products, specialized pro-resolving mediators), as well as their application in diabetic patients.

## 1. Introduction

Periodontitis is an inflammatory disease with a multifactorial etiology [1,2], which affects the tissues that serve the maintenance and functionality of the teeth on the dental arches. Periodontitis is characterized by the progressive loss of periodontal attachment, leading in time to bone destruction and even tooth loss; its negative end-points include a significant impairment of the functions of the stomatognathic system and of the patient’s quality of life [3]. The etiology of periodontitis is multifactorial. It is based on an aberrant immune function, a disorder that can be caused by multiple causes of an oral or systemic nature. Periodontal inflammation is triggered by periodontal pathogens, with a change in the oral ecosystem, in favor of gram-negative bacteria [4], which will aggravate the inflammatory reactions. Therefore, the occurrence of dysbiosis is incriminated in the onset of inflammation rather than the action of a single microorganism [5]. Periodontitis is the result of complex interactions between genetic and environmental factors, including the host’s inflammatory response to the microbial community (a bacterial biofilm) [6].

The evolution of periodontitis is, as has already been demonstrated and observed in current practice, nonlinear and heterogeneous. Periodontal disease often evolves by alternating between periods of activity, characterized by tissue loss, and periods of remission [7]. Furthermore, the evolution can be extremely different from one patient to another. Moreover, the evolution may suffer changes in the patient’s life, aging processes, the appearance and/or evolution of certain systemic pathologies [8], or epigenetic changes [9], which will alter the ability of an adequate immune response.

The dysregulated immune response is a hyperactive one, in which excessive inflammatory reactions will favor dysbiotic changes and the degradation of periodontal tissues [10]. Polymorphonuclear cells (PMNs) play an important role in this phenomenon through their hyper-functionality, which will lead to the excessive secretion of proinflammatory cytokines and chemokines. It is important that these proinflammatory mediators do not remain confined to the periodontal tissue but pass into the bloodstream, affecting distant organs and systems [10]. In addition, hyperactive PMNs can increase the production of oxygen radicals (ROS) [11], increasing oxidative stress, and can contribute to the recruitment and activation of osteoclasts, cells with an essential role in bone resorption [12]. Excessive endogenous ROS production is likely to contribute to periodontal tissue degradation [13], activating matrix metalloproteinases (MMPs) and stimulating bacterial proteinase activity [14], molecules involved in periodontal tissue degradation.

**Figure 1:**
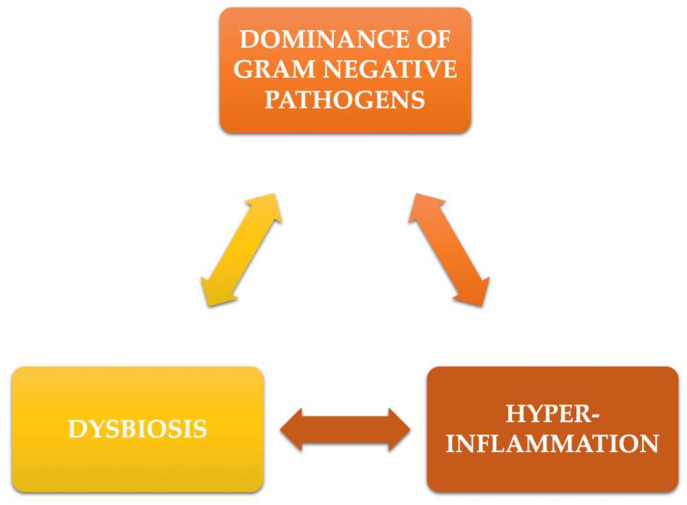
The vicious circle in periodontitis inflammation.

At the same time, negative influences on neutrophils have been observed, which include chemotaxis deficiency, phagocytosis disorders, respiratory explosion, and intracellular killing [15]. Such phenomena, together with the deficiency of leukocyte adhesion, will, in fact, cause an escalation of the inflammatory reactions. These events may be accompanied by the activation of Th-17 cells, which will try to compensate for the immune deficiencies already mentioned [16]. These processes will contribute to the creation of an even more dysbiotic environment, producing, in essence, a vicious circle that, clinically, will result in continuous loss of periodontal tissue (Figure 1).

The resolution of inflammatory phenomena is a complex, well-organized process that involves anti-inflammatory and anti-oxidant mediators. Anti-inflammatory mediators include lipoxins, maresins, protectins, or resolvins, the synthesis of which is signaled by pro-inflammatory mediators produced during inflammation [17,18]. Based on these findings, the concept of modulating the inflammatory response of the host was developed. These pharmacological strategies involved anti-inflammatory effects and, more recently, obtaining the resolution of inflammation.

Additionally, proinflammatory and anti-inflammatory reactions and processes can be marked by genetic factors, pathological factors (systemic diseases such as cardiovascular diseases [19], diabetes [20], or kidney diseases [21]), lifestyle factors (stress [22], smoking [23], diet [24], sedentary lifestyle [25]) or factors related to changes in dental arches (dental malpositions and incorrect treatments) [26]. Often, these factors can act simultaneously and exert different influences on different individuals [3], which contributes to the heterogeneity of periodontal disease.

Diabetes mellitus (DM) is a disease in which the body’s ability to produce or respond to the insulin hormone is affected [27]. The consequences are an abnormal metabolism of carbohydrates, as well as increased levels of glucose in the blood (hyperglycemia) and urine (glycosuria) [28]. DM is characterized by a number of important complications of a macro-vascular nature (coronary heart disease and stroke) or microvascular (nephropathy, retinopathy, neuropathy), periodontitis included [28,29]. Data from the literature suggest that metabolic changes in patients with type 1 diabetes are primarily due to dysfunction in insulin production. Type II diabetes is characterized by both innate and adaptive immune responses, which lead to the development of insulin resistance [30].

Diabetes, or, more exactly, the level of glycated hemoglobin (HbA1c), has become a descriptive factor in the current classification of periodontal diseases [31]. This fact generates the necessity of finding effective therapies for addressing patients with DM and periodontitis. Even if scaling and root planing (SRP), as part of periodontal treatment, has proven its beneficial role in improving HbA1c levels, new strategies have been developed, which also include means of inflammatory host response modulation [27]. Naturally, neither SRP nor anti-inflammatory and pro-resolving therapies are not indented as DM treatment by themselves. Still, if treating a local pathology, such as periodontitis, could also exert improvements on a systemic level, this would translate into a valuable bullet that resides at the core of interdisciplinarity and periodontal medicine.

The concept of host modulation therapy is not a new one, but it is continuously expanding. Its potential role in patients with periodontitis and systemic diseases still represents a rather un-charted area. Therefore, the aim of the present paper is to explore the known host response modulation therapies in the context of both periodontitis and DM presence and of their intertwined pathological mechanisms. The MEDLINE/PubMed, ISI Web of Science, Scopus, and Science Direct databases were accessed, and the following keywords were used: periodontitis, diabetes mellitus, host inflammatory response, inflammation, oxidative stress, host modulation therapy, inflammation resolution, and anti-oxidants. A total number of 6173 results were identified, of which the most important 124 articles in English, between 1992 and 2022, were analyzed. A two-stage screening process (titles and abstract, followed by full-text analysis) was performed by three independent reviewers (I.-G.S., S.T, and S.M.S.).

## 2. Pathophysiological Mechanisms in the Diabetes—Periodontitis Interaction

Diabetes may increase susceptibility to periodontal disease through mechanisms that include periodontal dysbiosis, the host’s inflammatory/immune response, and direct destruction of periodontal tissue. The essential role in these phenomena is represented by hyperglycemia, the production of advanced glycation end-products (AGE) and their receptors (RAGE), inflammation, and oxidative stress [32].

Shi et al. [33], in a longitudinal metagenomic analysis of the subgingival microbiome, showed that, among patients who developed periodontitis during follow-up, the transition from the normal microbiome to dysbiosis was higher in subjects with DM than in subjects without DM. Thus, it is assumed that diabetic patients are less tolerant of the presence of periodontal pathogens [33].

Xiao et al. [34], in an experimental murine study, demonstrated that mice with type 2 DM showed significant changes in microbiota composition compared to animals without DM. Moreover, an increase in the expression of interleukin 6 (IL-6) and the receptor activator of nuclear factor kappa-Β ligand (RANKL), molecules involved in bone resorption, was observed following infection with oral bacteria from DM mice to non- DM mice, compared to transfer from non-DM mice [34]. Thus, DM can increase the inflammatory response to oral bacteria. Kang et al. [35] showed that DM aggravated periodontal destruction in periodontitis induced by *Aggregatibacter actinomycetemcomitans*, with a significant increase in tumor necrosis factor alpha (TNF-α) expression and leukocyte infiltration.

In vitro, hyperglycemia, AGE, and *Porphyromonas gingivalis* lipopolysaccharides (LPS) demonstrated synergistic effects in modulating Toll-like receptor (TLR) expression, nuclear factor kB (NF-kB) activation, and proinflammatory cytokine production [36,37]. The AGE/RAGE system can induce an inflammatory response through NF-κB pathways in vitro in gingival fibroblasts [38] and increase the adhesion of co-cultured monocyte cells [39]. AGEs can accumulate in periodontal tissue under conditions of hyperglycemia, which can lead to collagen fibers damage [40].

The increased numbers but reduced function (chemotaxis and phagocytosis) of neutrophils in periodontal tissue have been reported in diabetes [41], which can aggravate periodontal destruction with poor defense against periodontopathogens. Poorly functioning neutrophils can enhance tissue damage without providing an effective defense against pathogens [42]. In patients with DM, neutrophils overexpress a key enzyme in the formation of neutrophil extracellular traps (NETs) [43]. The phenomenon of NETosis occurs, which promotes inflammatory status and inhibits the action of defense in periodontal sites [44].

In addition, epigenetic changes in periodontal tissues have been observed in diabetic patients; changes in the methylation of over 1000 genes have been reported in the healthy periodontium in animals with DM, with an overexpression of the proinflammatory molecules genes, which exacerbates the inflammatory status [45].

The main mechanisms by which periodontitis may influence the development of insulin resistance include (a) the passage of periodontal pathogens and their products into the bloodstream; (b) the spread of proinflammatory molecules that will increase systemic inflammatory status; (c) disruption in the intestinal microbiota balance and increased intestinal permeability, induced by swallowed periodontal bacteria [32].

Periodontal bacteria and their products have also been found at distant sites, such as atheroma plaques [46], lung tissue [47], or pancreas [48]. Diabetic patients are characterized by susceptibility to infections, the dissemination of circulating periodontal pathogens being favored [49,50]. These aspects are also facilitated by endothelial dysfunction and impaired microcirculation in patients with DM [32]. Moreover, periodontopathogens may alter the architecture of pancreatic β-islands, affecting insulin production [51,52], or may contribute to insulin resistance through their metabolic activity [53].

Periodontal bacteria can be swallowed, inducing intestinal dysbiosis [54]. The oral administration of *P. gingivalis* in a murine model of experimentally-induced DM generated intestinal dysbiosis, changes in intestinal metabolites, increased hepatic gluconeogenesis, and fasting hyperglycemia [55].

As already mentioned, the cytokines released during periodontal disease can reach the bloodstream. Periodontitis patients have been shown to have elevated blood levels of pro-inflammatory markers, such as C reactive protein (CRP), fibrinogen, IL-6, interleukin-1 (IL-1), and TNF-α [56]. These markers may promote insulin resistance [57], aggravating diabetic status, as well as its complications.

## 3. Agents Involved in the Modulation Therapy of the Host’s Inflammatory Response

**Figure 2:**
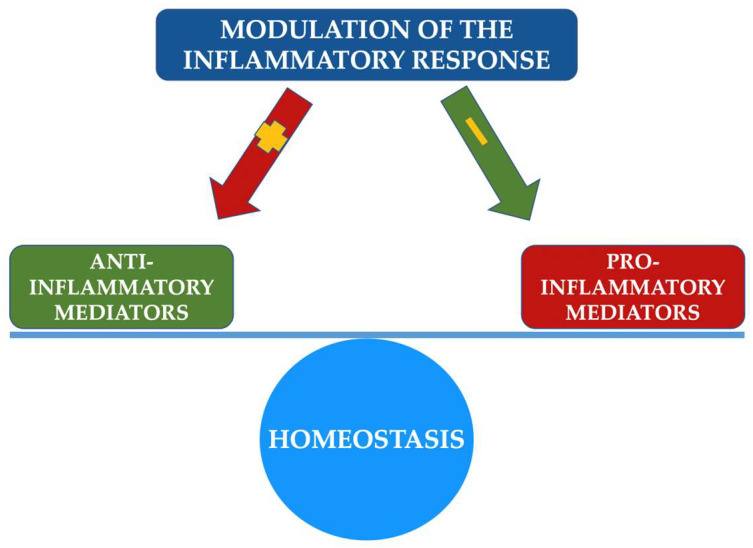
Synthesis of host inflammatory response therapy action pathways.

**Figure 3:**
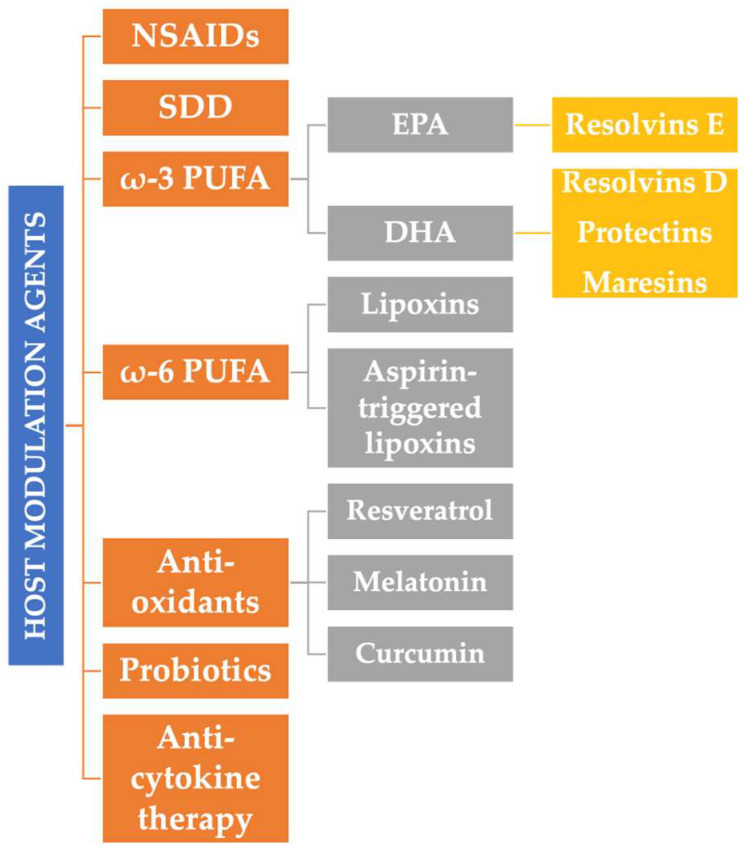
The main classes of substances used in the modulation therapy. NSAIDs: non-steroidal anti-inflammatory drugs; SDD: sub-antimicrobial doses of doxycycline; ω-3/6 PUFA: omega 3/6 polyunsaturated fatty acids; EPA: eicosapentaenoic acid; DHA: docosahexaenoic acid.

Host modulation involves a therapeutic concept that aims to change the state and/or function of the human body as a host in order to treat a disease. The modulation of the host’s inflammatory response in periodontal disease proposes a modification of the immune response, preventing or ameliorating periodontal destruction [58], with the help of anti-inflammatory, anti-oxidant, modulatory substances. In essence, modulation therapy aims to restore the balance between proinflammatory and anti-inflammatory factors, stopping the evolution of periodontitis and recreating an environment conducive to healing (Figure 2) [59]. Of course, this therapy comes as an adjunct form of periodontal treatment, the gold standard being maintained by periodontal mechanical debridement with scaling and root planing. The main classes of agents used in the modulation therapy of the host’s inflammatory immune response are synthesized in Figure 3.

### 3.1. Anti-Inflammatory Drugs

Among the first investigated drugs used in this form of therapy are non-steroidal anti-inflammatory drugs (NSAIDs), such as ibuprofen or indomethacin [60]. The use of NSAIDs was intended to block the cyclooxygenase (COX) pathway and arachidonic metabolism, leading to the blockade of prostaglandin E2 (PGE2) secretion. PGE2 is a proinflammatory molecule produced by different cell types (fibroblasts, PMN macrophages), following stimulation by lipopolysaccharides (LPS) and proinflammatory cytokines [58]. The effects of PGE2 include vasodilation, increased vascular permeability, and bone resorption [61].

The use of NSAIDs has been shown to be effective in periodontal treatment, decreasing inflammation and bone loss [62,63,64,65]. All of these beneficial effects persist; however, as long as the therapy is maintained, recurrence phenomena were observed after drug discontinuation. This would translate clinically into chronic treatment, but these drugs cannot be administered chronically due to their important side effects: gastrointestinal disorders [66], hepatic and renal toxicity [67], and pro-thrombotic effects [68]. Thus, the use of anti-inflammatory drugs is not a viable form of modulation therapy.

The use of statins, inhibitors of 3-hydroxy-3-methylglutaryl coenzyme A reductase (HMG-CoA reductase), was also investigated. These substances have proven extremely beneficial effects, inhibiting proinflammatory cytokines, reducing bacterial growth, disrupting the stability of the bacterial membrane, and increasing bacterial clearance. Clinically, they prevent bone resorption and promote bone neo-formation [69]. The major disadvantage of statins is that they generate their beneficial effects only during the therapeutic period, which causes them to be inadequate as a modulation therapy.

### 3.2. Anti-Cytokine Therapy

Anti-cytokine therapy uses substances such as monoclonal antibodies or receptor antagonists in order to block the action of pro-inflammatory cytokines. Infliximab (monoclonal antibody against TNF-α), etanercept (a soluble form of the TNF-α receptor), or anakinra (an IL-1 receptor antagonist) are examples of such drugs commonly administered in the treatment of rheumatoid arthritis. Their effectiveness in improving the periodontal status and reducing inflammation has been supported by a number of studies [70,71,72].

Similar to NSAIDs, the action of these drugs persists only during treatment. Moreover, they also have the disadvantage of generating adverse effects on the immune system [73]. In addition, they have a specific action that targets a certain mediator, which can make them ineffective in cases of complex inflammatory cascade [74].

### 3.3. Sub-Antimicrobial Doses of Doxycycline

To date, the only drug approved by the U.S. Food and Drug Administration (FDA) as a modulator of the host response in periodontitis is represented by sub-antimicrobial doses of doxycycline (SDD: Periostat^®^) (CollaGenex Pharmaceuticals, Inc., Newton, PA, USA) [75]. Periostat^®^ consists of 20 mg of doxycycline, administered twice a day for 3–9 months [76]. The effects of SDD include inhibition of MMPs, as well as the destruction of ROS, with reduced tissue proteinase activity by protecting the α1-proteinase inhibitor [77].

A number of studies have demonstrated the clinical and molecular efficacy of SDD [75,78,79,80]. Importantly, this form of therapy did not generate resistant microorganisms, and the observed beneficial effects persisted even after SDD cessation [81].

### 3.4. Specialized Pro-Resolving Mediators

Specialized pro-resolving mediators (SPM) are lipid molecules whose role is to inhibit and eliminate the stimulus which maintains tissue dyshomeostasis, as well as to promote local repair or regeneration phenomena [82]. SPM can be represented by lipoxins, protectins, resolvins, maresins, or annexins. SPM does not simply block inflammation, but counteracts the mechanisms of hyper-inflammation, promotes the clearance of inflammatory products, and stimulates the restoration of tissue architecture and functionality.

Two main classes of lipoxins have been identified: lipoxins (LX) and aspirin-triggered lipoxins (ATL). These molecules are endogenously generated lipid mediators with pleiotropic functions. They are synthesized in the late stages of inflammation from arachidonate (omega-6, ω-6 PUFA polyunsaturated fatty acids), cleaved from the cell membrane. Lipoxin-releasing cells include leukocytes, platelets, endothelial cells, epithelial cells, or periodontal stem cells [83].

Lipoxins interact with neutrophil receptors, inhibiting chemotaxis and the secretion of pro-inflammatory molecules. Moreover, in the presence of lipoxins, monocytes and macrophages exert the function of phagocytosis without secretion of pro-inflammatory interleukins [84]. A number of studies have shown a reduction in inflammatory phenomena in periodontitis following lipoxin administration [85,86].

Resolvins are metabolites of essential omega-3 polyunsaturated fatty acids (ω-3 PUFA) cleaved from the cell membrane—eicosapentaenoic acid (EPA) and docosahexaenoic acid (DHA). EPA metabolites form the resolvin E series (RvE), while DHA is the origin of the series that includes resolvins D (RvD), protectins, and maresines [87].

Resolvins, similar to lipoxins, suppress chemotaxis and neutrophil infiltration, decrease proinflammatory interleukin production, and induce neutrophil apoptosis. They are synthesized by cells such as PMN, vascular endothelial cells or mesenchymal stem cells [88]. Resolvins promote the elimination of periodontal infection, prevent its recurrence, promote the proliferation and differentiation of stem cells and even have the ability to reverse dysbiosis of the oral microbiome [89]. Resolvins have shown beneficial effects on bone resorption by suppressing RANKL or stimulating osteoprotegerin synthesis (OPG), inhibiting osteoclastogenesis [90,91,92], or enhancing the effects on periodontal ligament fibroblasts proliferation [93].

Maresins, derived from DHA, are macrophage mediators of inflammation. Their biosynthesis takes place, in particular, from M2 macrophages [94]. Maresins prevent the expression of proinflammatory cytokines by inhibiting the TLR4/mitogen-activated protein kinase (MAPK)/NFkB signaling pathway [95]. They also inhibit the phenotypic transformation of fibroblasts by inhibiting the extracellular signal-regulated kinase (ERK)/Smad signaling pathway. Moreover, maresins regulate the expression of detoxification enzymes and oxidoreductase by activating the Nrf-2 signaling pathway while reducing the production of reactive oxygen species by inhibiting the NFkB signaling pathway [96]. They have proven important roles in decreasing ROS [97], as well as in collagen fibrillogenesis and migration of osteoblasts, fibroblasts, and stem cells [98].

The ω-3 PUFAs, represented by EPA and DHA, are essential fatty acids. Their source is exogenic, with a recommended daily intake of 500 mg EPA and DHA for people without cardiovascular risk and 1 g for people with cardiovascular diseases [99]. Fish oil and some crustaceans are their main natural source [100]. PUFAs replace arachidonic acid in cell membranes and are used in the synthesis of thromboxanes, as well as, already mentioned, resolvins, proteins, and maresins [101].

EPA and DHA have the ability to alter the cellular functions of PMN, modulate lymphocyte proliferation, and increase the antioxidant capacity of the host. They inhibit the production of arachidonic acid metabolites by cyclooxygenase and lipoxygenase, thus reducing the synthesis of proinflammatory arachidonic acid metabolites [102].

The supplementation of non-surgical periodontal therapy with ω-3 PUFAs has been shown to be beneficial [103], especially when combined with low-dose aspirin (75–100 mg) [104,105]. In this case, aspirin has the role of acetylating and modifying the enzymatic function of cyclooxygenase-2, favoring the synthesis of resolvins E, D, and protectins [106]. The ω-3 PUFAs have also been shown to inhibit aggressive periodontopathogens such as *P. gingivalis* or *Prevotella intermedia* [107]. It also inhibits collagenase activity and suppresses the production of IL-1, IL-6, and TNF-α, together with the suppression of lipopolysaccharide activity [108]. Most studies have focused on an ω-3 PUFA dosage of 900–1500 mg per day for 3–6 months [105].

### 3.5. Probiotics

Probiotics are defined as live cultures of microorganisms that confer a health benefit to the host when administered in appropriate dosages [109]. Their use as adjuvant periodontal treatment was also investigated; they have proven both antimicrobial and immuno-modulatory effects through still incompletely elucidated mechanisms [110].

Different probiotic cultures have been shown to restore homeostasis of the oral microbiome, compatible with a healthy status, in a manner, however, effective only during therapy [111]. The antimicrobial activity of probiotics involves altered microbial signaling and the host’s subsequent immune response.

Lactobacilli have been shown to modulate the inflammatory response to periodontopathogens [112]. A number of studies have shown that *Lactobacillus* species can mediate the inflammatory response to *P. gingivalis* [113], lowering the levels of IL-1β, IL-6, and TNF-α.

Probiotic therapy reduced alveolar bone loss in mice with experimentally induced periodontitis [114]. Another study showed that lactobacilli were used as a vehicle to provide bacterial antigens that induce a protective immune response [115]. *Lactobacillus acidophilus* expresses FomA, an antigen of the outer membrane of *Fusobacterium nucleatum* [109]. *L. acidophilus* increased immunity against *P. gingivalis* and *F. nucleatum* as serum antibodies to immunoglobulins G and salivary immunoglobulins A and an increased response to the inflammatory cytokine IL-1β [116].

### 3.6. Other Substances

The influence of other substances with a potential modulating and anti-oxidant role was also investigated. Resveratrol has demonstrated anti-inflammatory [117] and antioxidant [118] properties. Resveratrol may inhibit osteoclast differentiation and activation. It suppresses IL-1β, IL-8, and monocyte chemoattractant-1 (MCP-1) in a manner independent of sirtuin 1 (Sirt1) [119]. Thus, resveratrol has multifunctional and beneficial effects, targeting inflammatory genes, osteoclast differentiation, and oxidant-related genes [120].

In an in vitro model, resveratrol inhibited the cytokines IL-1β, IL-6, IL-8, IL-12, and TNF-α, as well as nitric oxide production [121]. Ikeda et al. [122] demonstrated that a natural source of resveratrol inhibited periodontal degradation, associated with decreased oxidative stress and reduced osteoclast differentiation and activity in a murine model of experimental periodontitis. There are, however, a number of concerns about the poor bioavailability of resveratrol, partially associated with rapid urinary excretion [123].

Melatonin is an endogenous hormone that is released mainly by the pineal gland during night-time conditions [124]. Melatonin plays an important role in several biological processes, including immune responses, anti-inflammatory processes, bone homeostasis, and energy metabolism [124,125]. Moreover, it also generates antioxidant effects [126].

Another product, curcumin, has been shown to inhibit the activation of nuclear factor NF-κB, a molecule that stimulates a number of inflammatory markers, such as TNF-α [127,128]. Other research suggests that the anti-inflammatory mechanism may be due to blocking the metabolism of arachidonic acid, with the following phenomena: (a) selective inhibition in the synthesis of PGE2 and thromboxane; (b) inhibition of arachidonic acid metabolism by lipoxygenase; (c) elimination of generated free radicals and decreased inflammatory cytokine expression of IL-1β and IL-6 [129,130]. Curcumin also inhibits the release and regulation of several MMPs and reduces the release of many proteolytic enzymes, such as elastase, collagenase, and hyaluronidase, from activated macrophages [131,132]. Curcumin has proven effective as adjunctive therapy for periodontal disease in studies that have investigated various formulations: rinses and oral irrigation, gels, and even as a photosensitizing agent in periodontal photodynamic therapy [133].

## 4. Modulation of the Host Response in Patients with Diabetes Mellitus

The use of substances that can modulate the host inflammatory response was also investigated in the context of the concomitant presence of periodontitis and diabetes mellitus, especially type II DM. Below we present a series of data from the literature on the effectiveness of these methods in a pathological association which, as already mentioned, is characterized by demonstrated significant reciprocal influences.

### 4.1. Sub-Antimicrobial Doses of Doxycycline

Data on the use of SDD in patients with periodontitis and diabetes are quite limited. Gilowski et al. [134] conducted a study on 34 patients with type II DM, divided into two groups: the test group, which followed periodontal mechanical debridement (SRP) and SDD for 3 months, and the control group, which followed SRP and placebo. The investigated periodontal parameters, probing depth, periodontal clinical attachment level, and bleeding on probing showed improved values for both groups, but the benefit was only demonstrated for patients with moderate losses of periodontal attachment [134]. The authors also assessed the levels of MMP-8 in gingival crevicular fluid (GCF); it showed significant decreases only for the group with SDD. HbA1c analysis did not reveal significant differences between groups.

Engebretson and Hey-Hadavi [135] conducted a study of 45 patients with type II DM, diagnosed for more than 9 years, and untreated chronic periodontitis. The subjects received either SRP + SDD for three months, SRP + placebo or SRP + doxycycline in antimicrobial doses for 2 weeks. The only group that showed significant reductions in HbA1c was the group with SDD (a reduction of 0.9%).

Another study investigated GCF changes of MMP-9 and MMP-13, molecules involved in the destruction of collagen and alveolar bone, following the administration of SDD for three months, in patients with type II DM and periodontitis Stage 2, Grade B [136]. The authors noted that SRP and 20 mg of doxycycline exerted a more significant reduction in periodontal indices (probing depth, plaque index, gingival index), as well as in MMP-9 and MMP-13 than patients who received only SRP.

On the other hand, two systematic analyzes [137,138] failed to demonstrate significant improvements that SDD therapy could bring in addition to SRP alone in patients with DM and periodontitis. Another meta-analysis concluded that the addition of photodynamic therapy to the SRP + SDD regimen in diabetic patients could maximize beneficial outcomes in terms of periodontal parameters and HbA1c [139]. Indeed, the studies found in the literature are very heterogeneous; most of them were performed on small groups of patients and with a limited evaluation period (3 months).

### 4.2. Supplementation with ω-3 PUFAs

Most investigations of ω-3 PUFAs supplementation in diabetic patients have focused on patients with type II DM, a pathological form in which diet and lifestyle, in general, have a considerable influence [140]. The beneficial effects of ω-3 PUFA intake on metabolic profiles in patients with type II DM and obesity have been demonstrated [141].

The etiopathogenic phenomena of type II diabetes can be influenced by affected metabolic profiles, inflammation, and oxidative stress. There is evidence that PUFA can have anti-inflammatory effects and reduce oxidative stress [142]. In the light of these particularities, several studies evaluating the beneficial effect of ω-3 PUFA intake on metabolic parameters in patients with DM type II have been performed. Khalili et al. [143] observed that PUFA supplementation may generate favorable effects on glycemic factors, lipid profile, inflammatory markers, and body weight [143].

The ω-3 PUFAs supplementation was also investigated in patients with DM and periodontitis. Damaiyanti et al. [144] examined the effect of dietary supplements with 4 mL/kg or 16 mL/kg *Sardinella longiceps* fish oil on protection against periodontal damage resulting from the expression of MMP-8 and on tissue inhibitor of metalloproteinase 1 (TIMP-1) in Wistar mice with induced DM. TIMP levels are generally higher in healthy periodontal tissues than those affected by periodontal inflammation, which generates excessive MMP production [145]. The authors noted that the treatment group showed a significant reduction in MMP-8 expression and a marked increase in TIMP-1 expression; the best results were generated by the dose of 16 mL/kg *Sardinella longiceps* fish oil [144].

Castro Dos Santos has conducted a series of studies on the modulation of the inflammatory response from periodontitis in diabetic patients. SRP was performed, adding ω-3 fish oil (3 g) and low-dose aspirin (100 mg) for two months [146,147,148]. The authors proposed a protocol with changes during administration. After initial therapy, patients received ω-3 and aspirin for 2 months before SRP in order to evaluate clinical and immunological variables in the use of only ω-3 PUFA and low-dose aspirin without subgingival debridement. The authors demonstrated that the use of ω-3 PUFA and aspirin without SRP did not promote clinical and immunological changes in periodontitis. With the application of SRP, the additional benefits of host modulation therapy were significant [146]. This combination therapy also significantly reduced HbA1c (−0.51%). The authors later investigated the impact on quality of life in these patients that the proposed combination therapy could generate [148]. Oral Health Impact Profile (OHIP-14) was observed to decrease significantly after therapy, indicating improved quality of life.

Zare Javid et al. [149] investigated the effect of SRP combination with ω-3 PUFA and cranberry juice in patients with DM and periodontitis. Forty-one patients received either SRP, SRP + ω-3 (1 g twice, daily), SRP + cranberry juice (200 mL/day), or SRP + cranberry juice enriched with ω-3 PUFA (200 mL juice with 1 g ω-3 PUFA) for two months. Pre- and post-therapeutic glycemia and HbA1c, lipid profile, periodontal probing depth, and anthropometric indices were investigated. The SRP + cranberry juice enriched with ω-3 PUFA therapy generated the most favorable results, improving the probing depth, as well as the HbA1c and high-density lipoprotein (HDL)-cholesterol values [149].

### 4.3. Other Modulation Therapies

As already mentioned, the imbalance between oxidant and antioxidant status has an extremely important role in the pathology of both periodontitis and diabetes. Thus, an attempt was made to develop therapies that intervene in this etiopathogenic aspect as well.

Zare Javid et al. [150] conducted a placebo-controlled study in 43 patients with DM and periodontitis who followed either SRP and resveratrol (240 mg/day) or SRP and placebo for four weeks. IL-6, TNF-α, total serum antioxidant capacity (TAC), and loss of clinical attachment (CAL) were assessed at baseline and at the end of therapy. The authors demonstrated that the beneficial effect of resveratrol supplementation was reflected only in IL-6 decreases.

Several investigations have reported the positive influences of periodontal mechanical debridement therapy supplemented with melatonin. Mizutani et al. [124], in a recent meta-analysis, observed the beneficial effects on periodontal parameters of melatonin supplementation in patients with DM and periodontitis. Bazyar et al. [151] observed that SRP and melatonin therapy (6 g/day, 8 weeks) significantly improved periodontal parameters, as well as levels of IL-6 and high-sensitivity C-reactive protein (hs-CRP). In an interventional study in patients with type II DM and periodontitis, we investigated the effects of melatonin supplementation (3 g/day for 8 weeks) and SRP on probing depth, periodontal attachment loss, and bleeding on probing. These parameters showed significant improvements compared to subjects in which only SRP was performed [152]. Moreover, this form of therapy has also generated significant improvements in HbA1c.

The effects of curcumin were evaluated in a study on an experimentally-induced DM and periodontitis murine model. Deng et al. [153] investigated the impact of a modified form of curcumin (CMC 2.24), administered by oral gavage for 3 weeks, by computed tomography analysis, as well as on the inflammatory reaction. CMC 2.24 has been shown to decrease macrophage accumulation, regulate PMN chemotaxis, reduce levels of MMPs and proinflammatory cytokines, and significantly increase the action of resolvin RvD1 [153].

Other substances have also been the subject of isolated investigations. Propolis has been shown to improve periodontal status in both healthy patients [154] and in those affected by type II DM [155]. Moreover, the administration of 400 mg of propolis per day for 6 months, together with SRP, significantly reduced the HbA1c value. The administration of 2 g of ginger twice daily, for 8 weeks, in addition to SRP, in patients with DM and periodontitis, resulted in significant decreases in periodontal parameters (plaque index, probing depth, loss of attachment, and bleeding on probing), but also of TNF-α, IL-6, hs-CRP, superoxide dismutase (SOD), catalase (CAT), and glutathione peroxidase (GPx) [156].

## 5. Conclusions and Future Directions

The principles and substances addressed in modulating the host response have followed numerous changes and diversifications since the advent of this form of therapy, both in systemically healthy patients and, in the particular case of this paper, in diabetic subjects. Non-steroidal anti-inflammatory drugs have proven, through their limited efficacy and important side effects, not to be a viable form of modulation therapy. Though generating significant reductions in inflammatory products with beneficial effects in patients with DM, sub-antimicrobial doses of doxycycline have recently raised a number of concerns regarding the safety of long-term therapy.

An interesting perspective is given by the use of ω-3 PUFAs, by producing specialized pro-resolving mediators, which are able to stop the hyper-inflammatory process and promote periodontal healing, with effects on glycemic control and lipid profile. As we have seen, these effects can be potentiated by adding low-dose aspirin. A number of isolated studies have also proposed the use of substances such as propolis, melatonin, resveratrol, or curcumin as potential modulators of inflammation, but the data are, for the time being, insufficient to certify their absolute benefit in patients with periodontitis and DM.

Probably, the most important practical issue regarding the host response modulation therapy involves patient compliance. Most of the substances investigated require administration once or twice a day, for extended periods of 3, 6, or even 9 months, which can lead to an incomplete continuation of therapy. It becomes necessary to design formulations that reduce the intake frequency and duration.

Another aspect worth considering is that, in general, DM patients are already undergoing various forms of therapy for diabetes at the time of enrollment in interventional studies. Most studies do not specify the followed therapeutic form nor the potential bias factor that derives from its potential influences. In addition, there are insufficient data to clearly favor a form of therapy on their long-term effects, maintenance, and stability. Thus, studies that go beyond these limitations are needed.

## Data Availability

Not applicable.
